# Clinical evaluation of cold plasma surface activation using different plasma systems on early implant stability: a randomized controlled within-patient trial

**DOI:** 10.1186/s12903-026-08679-8

**Published:** 2026-05-30

**Authors:** Aykut Can Balkanlıoğlu, Izzet Acikan

**Affiliations:** Department of Oral and Maxillofacial Surgery, Faculty of Dentistry, Kahramanmaras Sutcu Imam University, Avsar street, Onikisubat, Kahramanmaras, 46060 Turkey

**Keywords:** Cold plasma, Dental implant, Implant stability, ISQ, Osseointegration, Randomized controlled trial, Within-patient design

## Abstract

**Background:**

Early implant stability is a relevant surrogate during the transition from mechanical primary stability to biologically mediated secondary stability. Cold plasma activation may improve titanium surface readiness, but direct clinical comparisons remain limited.

**Methods:**

This prospective, single-center, randomized, within-patient controlled trial compared untreated control implants with three pre-insertion plasma protocols: vacuum plasma, argon jet plasma, and cold atmospheric plasma. Eleven patients received 44 implants. One patient/four implants was excluded for insufficient primary stability. The final analysis included 40 implants in 10 patients, each contributing one implant per group. Implant sites were not anatomically mirrored. Plasma was applied for 60 s before placement. Implant stability quotient (ISQ) was recorded at baseline and at 14, 28, and 56 days. A nested linear mixed-effects model was used for statistical inference.

**Results:**

All analyzed implants remained in situ throughout follow-up, and no adverse events were recorded. Baseline ISQ values were comparable across groups (70.6–70.9), without establishing anatomical equivalence. Control implants showed a transient ISQ decrease at day 28, whereas plasma-treated implants showed increasing ISQ values. The group-by-time interaction was significant (likelihood-ratio chi-square(9) = 188.73, *p* < 0.001). At day 56, model-based mean differences versus control were + 10.0 ISQ units for vacuum plasma, + 8.9 for argon jet plasma, and + 8.3 for cold atmospheric plasma (all *p* < 0.001). Sensitivity analyses preserved the association direction.

**Conclusions:**

Cold plasma activation was associated with a more favorable early ISQ trajectory than untreated implants during the first 8 weeks. Given the small, retrospectively registered, site-heterogeneous, ISQ-based design, larger prospectively registered, site-standardized trials are warranted.

**Trial registration:**

Retrospectively registered at ClinicalTrials.gov, NCT07348770. First submitted: 11 December 2025; first posted: 16 January 2026; latest update posted: 11 May 2026.

**Supplementary Information:**

The online version contains supplementary material available at 10.1186/s12903-026-08679-8.

## Background

The long-term success of dental implants depends on the effectiveness of osseointegration, which is defined as the formation of a direct and functional connection between the implant and the surrounding vital bone [1]. While primary stability is achieved through mechanical interlocking of the implant with bone at the time of placement, secondary stability is gained as a result of biological processes related to new bone formation and remodeling [2]. The implant stability quotient (ISQ), measured by resonance frequency analysis (RFA), allows objective monitoring of this dynamic process [3]. Clinical studies have demonstrated that implant stability does not increase linearly; instead, a transient decrease known as the stability dip occurs between 2 and 4 weeks after surgery, followed by a subsequent increase associated with secondary stability [4, 5]. Reducing stability loss during this critical period is particularly important for the safety of accelerated rehabilitation and early loading concepts [6].

Implant surface topography is one of the most influential factors affecting the speed and quality of early osseointegration [7]. Moderately rough titanium surfaces are known to provide a higher bone-to-implant contact (BIC) percentage and stronger mechanical anchorage compared with smooth surfaces [7, 8]. In addition to surface topography, surface chemistry also plays a decisive role; high surface energy and hydrophilicity enhance early protein adsorption and osteoblast adhesion, thereby accelerating secondary stability [9]. Studies on hydrophilic modified SLA surfaces have demonstrated higher stability values during the early healing phase and a shorter duration of the stability dip [10].

However, titanium surfaces undergo biological aging over time due to the adsorption of hydrocarbon compounds from the atmosphere. This process leads to a reduction in surface energy and hydrophilicity, consequently decreasing the adhesion capacity of osteogenic cells to the surface [11]. Both in vitro and in vivo studies have shown that osteoconductivity significantly decreases as the post-manufacturing storage period increases [9–11].

To overcome these drawbacks, various surface reactivation methods have been developed. Ultraviolet photofunctionalization can decompose the hydrocarbon layer and render the surface superhydrophilic; however, integration of this approach into routine clinical practice may be challenging in some settings [12–14]. In recent years, cold atmospheric plasma and argon plasma applications have emerged as practical and rapid surface activation methods [15–17]. Plasma is a partially ionized gas composed of ions, radicals, and excited atoms; when interacting with titanium surfaces, it removes hydrocarbon contamination, increases hydrophilicity, and enhances surface energy [17, 18].

X-ray photoelectron spectroscopy analyses have confirmed that plasma treatment significantly reduces carbon content on titanium surfaces while increasing surface oxygen levels [18]. Experimental models have demonstrated increased protein adsorption, osteoblast adhesion, and early differentiation markers on plasma-treated surfaces [19, 20]. In addition, plasma has been reported to reduce biofilm formation on implant surfaces due to antimicrobial effects; this phenomenon has been observed on both titanium and barrier membrane materials [21–24].

Animal studies have shown that implants pretreated with cold plasma exhibit significantly increased BIC and bone volume fraction at 2–4 weeks, with similar effects reported across different plasma systems [18, 25–27]. In vivo canine models have also demonstrated that rough titanium implants activated with argon plasma achieve early osseointegration performance comparable to that of commercially available hydrophilic surfaces [28]. In human histological studies, plasma activation has been shown to significantly increase BIC values at 4 weeks [17].

Current clinical investigations report higher early ISQ values in plasma-treated implants compared with control groups, with attenuation of the early stability dip pattern [29, 30]. However, the number of clinical studies directly comparing different plasma systems—vacuum plasma, argon plasma jet, and atmospheric cold plasma—remains limited [13, 29–31]. Therefore, a clinical comparison of the effects of different plasma technologies on implant stability is scientifically warranted. In line with this cautious translational approach, a recently published exploratory randomized trial further indicates that small, biologically driven dental clinical studies should be interpreted as hypothesis-generating rather than as definitive evidence [32].

The aim of this study was to evaluate the effect of cold plasma surface activation on the early stability of dental implants and to explore whether different plasma application systems result in comparable clinical outcomes under a within-patient controlled design.

The null hypothesis was that cold plasma surface activation would not influence early implant stability and that no differences would be observed among the different plasma application systems.

## Materials and methods

### Study design, setting, and registration

This study was conducted as a prospective, single-center, randomized controlled clinical trial with a within-participant, site-level parallel allocation design and blinded outcome assessment. The trial was performed at the Department of Oral and Maxillofacial Surgery, Faculty of Dentistry, Kahramanmaraş Sütçü İmam University, Kahramanmaraş, Turkey. The manuscript was prepared in accordance with the CONSORT 2010 recommendations for randomized clinical trials, where applicable.

Each participant received four clinically indicated implants, and the four intervention protocols were allocated to different implant sites within the same patient. This design was chosen to reduce inter-individual biological variability. However, implant positions were determined according to prosthetic rehabilitation needs rather than predefined contralateral or mirrored anatomical sites. Therefore, the study should be regarded as a within-participant, site-level randomized parallel allocation trial, not as a strict split-mouth or crossover trial.

Blinding was limited to outcome assessment. The surgeon could not be blinded because the plasma devices and application procedures were visibly different. The assessor who recorded resonance frequency analysis and implant stability quotient values was blinded to treatment allocation. Because no sham plasma procedure was used, complete participant blinding could not be ensured. Accordingly, the correct masking definition is single masking of the outcome assessor.

The trial was retrospectively registered at ClinicalTrials.gov after study completion under the identifier NCT07348770. The current registry record lists the study as first submitted on 11 December 2025 and first posted on 16 January 2026, with the latest update posted on 11 May 2026. The updated record describes the study as an interventional randomized trial with parallel assignment and single masking of the outcomes assessor, with 11 enrolled participants and study dates from 24 February 2025 to 9 September 2025.

No clinical protocol change was implemented after recruitment, surgery, or follow-up. The inconsistency between earlier registry terminology and the actual study design was related to retrospective registration wording and data-entry classification, not to a post-recruitment protocol amendment. The updated public registry has been aligned with the implemented protocol and no longer describes the study as crossover, double-blind, or a strict mirrored split-mouth trial. Retrospective registration is acknowledged as an important methodological limitation.

### Ethics approval and consent to participate

Ethical approval was obtained from the Ethics Committee of Clinical Research of Harran University Faculty of Medicine (Approval No: HRÜ/25.03.28). All participants received oral and written information about the study and provided written informed consent before participation. The study was conducted in accordance with the Declaration of Helsinki.

### Participants and calibration

Before study initiation, a calibration meeting was held with the surgical and research team to standardize operative procedures, plasma application, RFA/ISQ measurement, follow-up scheduling, and data recording. Written standard operating procedures were used throughout the study. Operational calibration was performed; however, formal intra-observer repeatability statistics were not prospectively recorded.

A single blinded outcome assessor performed all RFA measurements using the same Osstell device and the same SmartPeg type compatible with the implant system. Measurements were made in two directions and averaged. This approach was intended to reduce inter-examiner variability.

### Eligibility criteria

Inclusion criteria were age 18 years or older, edentulous areas requiring fixed prosthetic rehabilitation supported by at least four implants, good to moderate oral hygiene, ability to attend follow-up visits, and voluntary willingness to participate.

Exclusion criteria were refusal to participate, need for alveolar ridge augmentation, systemic conditions that could impair wound healing, uncontrolled diabetes mellitus, immunodeficiency, ongoing chemotherapy or radiotherapy, previous radiotherapy to the head and neck region, substance or alcohol abuse, untreated periodontal disease, heavy smoking (> 10 cigarettes/day), age below 18 years, untreated local pathology at the surgical site, and failure to achieve protocol-defined primary stability (insertion torque < 45 Ncm). Because heavy smokers were excluded, included smokers were light smokers.

### Surgical procedures and interventions

All surgical procedures were performed under local anesthesia using 4% articaine with 1:100,000 epinephrine. Periodontal and restorative needs were completed before surgery. Patients rinsed with 0.2% chlorhexidine gluconate for 60 s before surgery. Full-thickness mucoperiosteal flaps were elevated, and osteotomies were prepared under copious sterile saline irrigation according to the manufacturer’s standard protocol unless a site-specific adaptation was required.

Each analyzed patient received four Medentika QuattroCone implants (Straumann Group, Germany). One implant per patient was allocated to each of the four study arms. Implant length (7, 9, 11, or 13 mm) and diameter were selected according to local anatomy, available bone height and width, mesiodistal restorative space, and the manufacturer’s recommendations. Implant dimensions were therefore clinical anatomy-driven variables, not randomized study factors.

Randomization was performed using an opaque sealed-envelope method. For each patient, an allocation sequence ensuring one assignment to each treatment group was prepared by a surgical assistant who was not involved in outcome assessment. After osteotomy preparation and immediately before the surface treatment step, the corresponding site envelope was opened and the assigned intervention was applied. This process preserved allocation concealment until the treatment assignment step.

Control group: implants were inserted without additional surface treatment.

Vacuum plasma group: implants were treated using a plasma activation device (Plasma Active, Point Implant, Germany). Implants were placed in a sterile low-pressure plasma chamber operating at 5–10 Torr and exposed to non-thermal cold plasma for 60 s immediately before placement.

Argon jet plasma group: implants were treated using a Piezobrush plasma system (Relyon Plasma GmbH, Regensburg, Germany) equipped with an argon gas module. Plasma was applied circumferentially for 60 s while the implant was mounted on a sterile rotating holder connected to a physiodispenser handpiece.

**Fig. 1:**
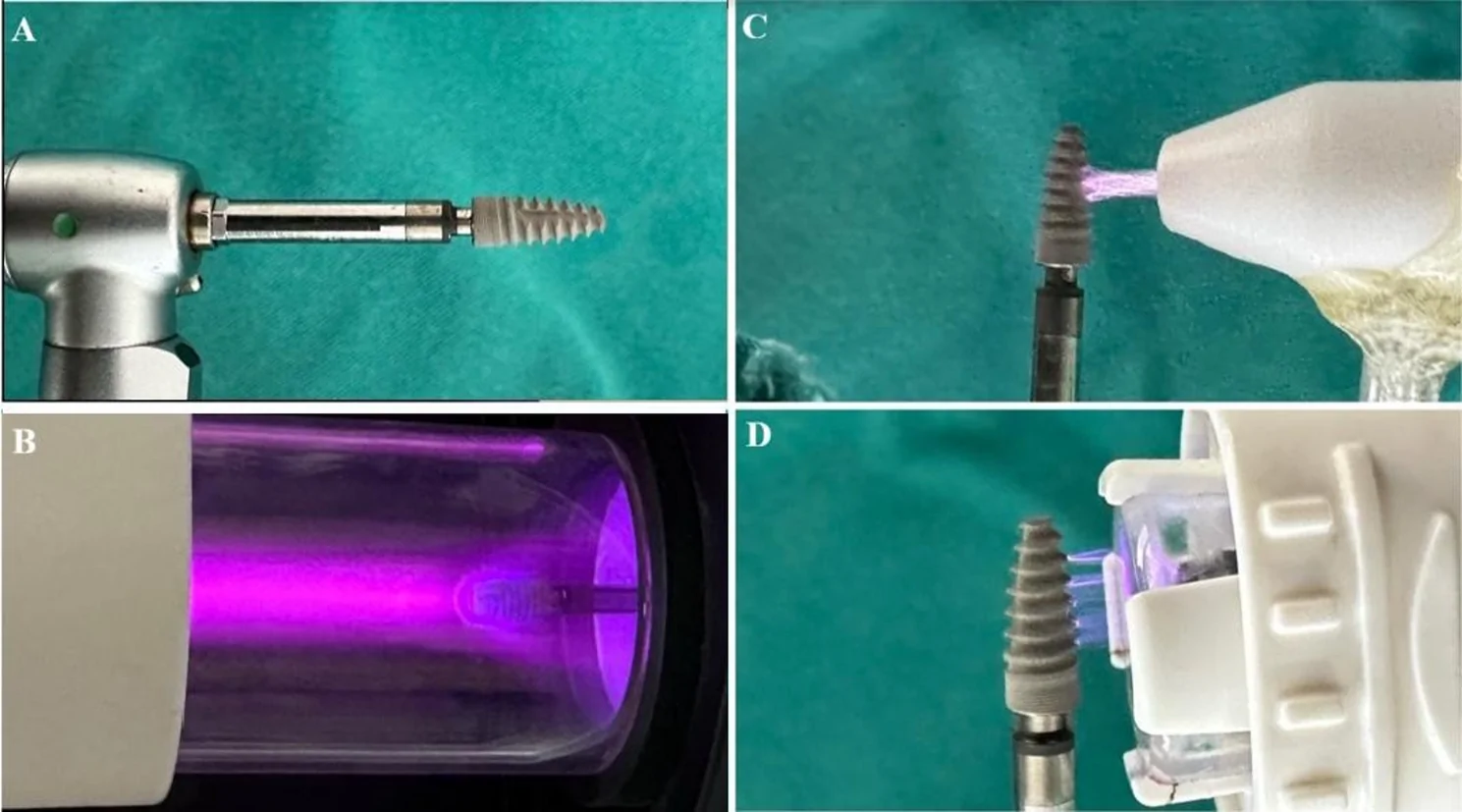
Chairside cold plasma surface activation protocols used in the study. **A** Control: implant placed without additional surface activation. **B** Vacuum plasma activation in a low-pressure plasma chamber immediately before placement. **C** Argon jet plasma application for 60 s while rotating the implant. **D** Cold atmospheric plasma activation using ambient air at atmospheric pressure for 60 s under sterile conditions

Cold atmospheric plasma group: implants were treated using the Piezobrush plasma system with the metal module, generating cold plasma from ambient air at atmospheric pressure. Plasma was applied circumferentially for 60 s while the implant was rotated under sterile conditions (Fig.1).

All implants were placed using a one-stage protocol, and healing abutments were connected during the same session. A minimum insertion torque of 45 Ncm was targeted as the protocol-defined threshold for adequate primary stability. Undersized osteotomy was not a group-specific intervention. It was used only once in the entire analyzed dataset, in one control implant in one patient, as a local bone-density-driven surgical adaptation. It was not applied to any implant in the vacuum plasma, argon jet plasma, or cold atmospheric plasma groups. Because it occurred only once, it was reported descriptively rather than included as a covariate in the mixed model.

Postoperative care included systemic antibiotics when clinically indicated according to the local protocol (amoxicillin-clavulanic acid 1 g twice daily or clindamycin 300 mg three times daily in penicillin-allergic patients for 7 days), ibuprofen 400 mg three times daily for 3 days as needed, and 0.2% chlorhexidine mouth rinse twice daily for 7 days. Sutures were removed after 7 days.

### Assessment of implant stability

Implant stability was assessed using RFA with the Osstell ISQ device (Osstell AB, Gothenburg, Sweden). SmartPeg Type 38 transducers compatible with the implant system were attached to the internal implant connection. Measurements were recorded in mesiodistal and buccolingual directions, and the arithmetic mean was used as the implant-level ISQ value. Measurements were performed immediately after placement (day 0), and at 14, 28, and 56 days postoperatively. ISQ values range from 1 to 100; values of approximately 70 or higher are commonly interpreted as high stability, whereas values in the 60–69 range indicate moderate stability [1].

### Outcomes

The primary outcome was time-dependent change in ISQ across four time points and four treatment groups. The secondary outcomes were implant survival during the 56-day observation period and the occurrence of local or systemic adverse events.

### Sample size and power

An exploratory a priori implant-level power calculation was performed using G*Power 3.1. Based on previous studies of plasma-modified implant surfaces, a medium effect size (Cohen’s f = 0.30), alpha = 0.05, power = 0.80, repeated-measure correlation = 0.50, and sphericity correction = 1.00 suggested a minimum of 32 implants in a two-way repeated-measures framework. The final analyzed sample contained 40 implants; however, because implants and repeated measurements were clustered within patients, the effective inferential sample was closer to the number of patients than to the raw implant count. Therefore, the study should be regarded as exploratory and hypothesis-generating rather than confirmatory.

### Statistical analysis

All analyses used the mean of the mesiodistal and buccolingual ISQ values. Descriptive statistics are presented as mean +/- standard deviation for continuous variables and as counts for categorical variables. Because the design generated multiple observations per implant and multiple implants per patient, linear mixed-effects modelling was used as the principal inferential framework.

Analyses were performed in Python 3.13.5 using statsmodels 0.14.6 and SciPy 1.17.0; data handling used pandas 2.2.3. Mixed-effects models were fitted by maximum likelihood (ML, not REML) because the principal group-by-time term was evaluated by likelihood-ratio comparison of nested fixed-effect models. Fixed-effect contrasts were interpreted using model-based Wald estimates, whereas the group-by-time interaction was evaluated using likelihood-ratio testing. Given the small patient-level sample (10 analyzed patients), all p values were interpreted as exploratory measures of association rather than confirmatory efficacy tests.

The main mixed model included treatment group, time, and the group-by-time interaction as fixed effects. Patient identity was specified as a random intercept, and implant nested within patient was specified as a variance component to account for repeated observations from the same implant. The model can be summarized as ISQ ~ group * time + (1 | patient) + (1 | patient: implant). Conditional on these random effects, residual errors were assumed independent with homogeneous variance; no unstructured residual covariance was fitted because of the small sample. The control group and day 0 were used as reference categories. Model fit was summarized using log-likelihood, -2 log-likelihood, and Akaike Information Criterion.

The intraclass correlation coefficient (ICC) was calculated from an ML null mixed model to quantify clustering under the same modelling framework. In this balanced dataset, the patient-level ICC was < 0.001, whereas the implant-level repeated-measure component accounted for approximately 0.215 of variance. These results indicated low between-patient mean variance in this small dataset but confirmed non-independence of repeated observations within implants; therefore, mixed modelling remained appropriate to avoid treating clustered observations as fully independent.

Clinically relevant pairwise comparisons were estimated from the model. Plasma-treated groups were compared with control at each postoperative time point. Pairwise comparisons among plasma systems were interpreted cautiously and adjusted within time point using Holm’s method. To address residual site-level confounding without overloading the primary model, two sensitivity analyses were added after review: a conservative one-control-implant deletion series and an exploratory jaw-adjusted LMM. In the deletion series, each control implant was removed one at a time and the main ML LMM was refitted; because undersized osteotomy occurred only once and only in the control group, this sensitivity series necessarily includes deletion of the undersized control implant while also testing whether the result depended on any single control implant. In the jaw-adjusted sensitivity model, jaw location was added as a fixed effect. These analyses were interpreted cautiously as robustness checks and were not treated as definitive covariate-adjusted evidence. Statistical significance was set at *p* < 0.05, but interpretation emphasized direction, magnitude, and consistency rather than isolated p values.

## Results

### Study population and site characteristics

Fifteen patients were assessed for eligibility. Four patients were excluded before enrollment because they did not meet the inclusion criteria. Eleven patients were enrolled, matching the actual enrollment reported in the updated ClinicalTrials.gov record. The public registry represents the enrolled/treated cohort (11 participants and 44 implants). For the stability analysis reported in this manuscript, one patient with four implants was excluded intraoperatively because insertion torque remained below the protocol-defined threshold of 45 Ncm. The final analyzed dataset therefore included 10 patients, 40 implants, and 160 ISQ observations.

The enrolled cohort included 5 males and 6 females, with a mean age of 52.4 years (range, 35–68 years). Three enrolled participants were light smokers; heavy smokers were excluded by protocol. In the analyzed sample, each treatment group contained 10 implants. The distribution of jaw location, implant length, smoking exposure, and undersized osteotomy by group is shown in Table 1. Jaw location and implant dimensions were not identical across groups because implant sites were dictated by clinical rehabilitation needs. Undersized osteotomy occurred only once, in one control implant, and did not occur in any plasma group.

**Table 1:** Baseline site and procedural characteristics by treatment group

Group	Implants (*n*)	Maxilla / Mandible	Implant length distribution (mm)	Smoker / Non-smoker implants	Undersized osteotomy, yes/tota
Control	10	4 / 6	7:2, 9:3, 11:3, 13:2	3 / 7	1 / 10
Vacuum Plasma	10	5 / 5	7:2, 9:6, 11:1, 13:1	3 / 7	0 / 10
Argon Jet Plasma	10	5 / 5	7:1, 9:1, 11:7, 13:1	3 / 7	0 / 10
Cold Atmospheric Plasma	10	4 / 6	7:1, 9:4, 11:5, 13:0	3 / 7	0 / 10

Values are counts unless otherwise stated. Smoking status is patient-level but is presented at implant level because each analyzed patient contributed one implant to each group. Undersized osteotomy was used in only one control implant in one patient and was not used in any plasma-treated implant

### Clinical outcomes and ISQ trajectory

All 40 analyzed implants remained in situ throughout the 56-day follow-up. No implant loss, local complication, systemic complication, or adverse event was recorded during the observation period.

Baseline ISQ values were numerically similar across groups: 70.6 +/- 1.5 in the control group, 70.9 +/- 1.6 in the vacuum plasma group, 70.7 +/- 1.3 in the argon jet plasma group, and 70.6 +/- 1.4 in the cold atmospheric plasma group. This similarity indicates comparable insertion-time mechanical stability, but it does not prove anatomical or biological equivalence of implant sites because ISQ does not capture all local bone-density, jaw-location, or site-condition differences.

**Fig. 2:**
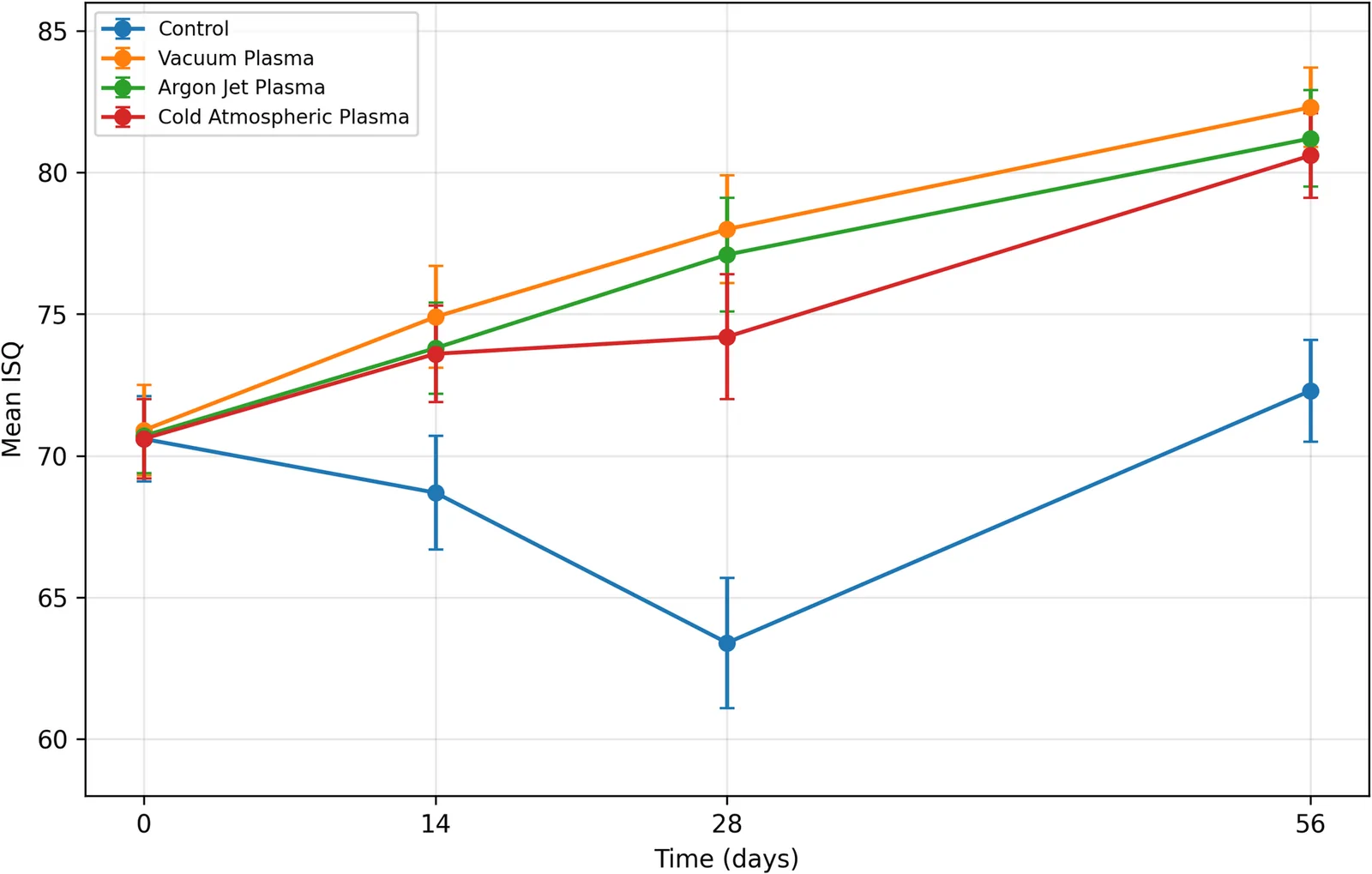
Time-dependent changes in implant stability (ISQ) across the study groups. Mean implant stability quotient (ISQ) values with standard deviation bars measured at days 0, 14, 28, and 56 in the Control, Vacuum Plasma, Argon Jet Plasma, and Cold Atmospheric Plasma groups

At day 14, mean ISQ values were higher in all plasma-treated groups than in the control group. At day 28, the control group showed its lowest mean ISQ value (63.4 +/- 2.3), whereas plasma-treated groups maintained increasing mean values. At day 56, all groups showed ISQ values in the high-stability range, but plasma-treated implants remained higher than untreated controls. Mean values are shown in Table 2; Fig. 2.

**Table 2:** Mean ISQ values by treatment group and follow-up time

Group	Day 0	Day 14	Day 28	Day 56
Control	70.6 ± 1.5	68.7 ± 2.0	63.4 ± 2.3	72.3 ± 1.8
Vacuum Plasma	70.9 ± 1.6	74.9 ± 1.8	78.0 ± 1.9	82.3 ± 1.4
Argon Jet Plasma	70.7 ± 1.3	73.8 ± 1.6	77.1 ± 2.0	81.2 ± 1.7
Cold Atmospheric Plasma	70.6 ± 1.4	73.6 ± 1.7	74.2 ± 2.2	80.6 ± 1.5

Values are mean +/- standard deviation. Each treatment group contained 10 implants, with one implant contributed by each analyzed patient

### Mixed-effects analysis and clustering

The ML null mixed model showed a patient-level ICC of < 0.001 and an implant-level repeated-measure ICC of 0.215 (approximately 0.22). In other words, between-patient mean variance was minimal in this balanced dataset, but repeated measurements from the same implant accounted for approximately 21.5% of total variance. The full mixed model converged and had a log-likelihood of -294.15, -2 log-likelihood of 588.30, and AIC of 626.3.

The group-by-time interaction was significant (likelihood-ratio chi-square(9) = 188.73, *p* < 0.001), indicating that the ISQ trajectory differed among groups. Model-based contrasts versus control were significant for all plasma-treated groups at days 14, 28, and 56 (Table 3). The largest difference versus control occurred at day 28 for vacuum plasma (+ 14.6 ISQ units). At day 56, differences versus control were + 10.0, + 8.9, and + 8.3 ISQ units for vacuum plasma, argon jet plasma, and cold atmospheric plasma, respectively.

**Table 3:** Model-based contrasts between plasma-treated groups and control

Time	Contrast	Estimated difference	95% CI	*p* value
Day 14	Vacuum Plasma - Control	6.2	4.7 to 7.7	< 0.001
Day 14	Argon Jet Plasma - Control	5.1	3.6 to 6.6	< 0.001
Day 14	Cold Atmospheric Plasma - Control	4.9	3.4 to 6.4	< 0.001
Day 28	Vacuum Plasma - Control	14.6	13.1 to 16.1	< 0.001
Day 28	Argon Jet Plasma - Control	13.7	12.2 to 15.2	< 0.001
Day 28	Cold Atmospheric Plasma - Control	10.8	9.3 to 12.3	< 0.001
Day 56	Vacuum Plasma - Control	10.0	8.5 to 11.5	< 0.001
Day 56	Argon Jet Plasma - Control	8.9	7.4 to 10.4	< 0.001
Day 56	Cold Atmospheric Plasma - Control	8.3	6.8 to 9.8	< 0.001

Estimated differences are in ISQ units from the linear mixed-effects model. Positive values indicate higher ISQ in the plasma-treated group than in control. All listed p values were < 0.001

Comparisons among plasma systems should be interpreted cautiously because the trial was not powered or site-standardized for plasma-to-plasma superiority. At day 28, cold atmospheric plasma showed lower mean ISQ values than vacuum plasma and argon jet plasma after within-time multiplicity adjustment. By day 56, adjusted plasma-to-plasma differences were not statistically significant (Table 4).

**Table 4:** Key plasma-to-plasma exploratory contrasts

Time	Plasma-system contrast	Estimated difference	Holm-adjusted *p* value
Day 28	Vacuum Plasma - Argon Jet Plasma	0.9	0.227
Day 28	Vacuum Plasma - Cold Atmospheric Plasma	3.8	< 0.001
Day 28	Argon Jet Plasma - Cold Atmospheric Plasma	2.9	< 0.001
Day 56	Vacuum Plasma - Argon Jet Plasma	1.1	0.279
Day 56	Vacuum Plasma - Cold Atmospheric Plasma	1.7	0.067
Day 56	Argon Jet Plasma - Cold Atmospheric Plasma	0.6	0.420

Estimated differences are in ISQ units. Plasma-to-plasma comparisons were exploratory and not the primary objective of the study

### Jaw-stratified descriptive analysis

Because implant sites were not anatomically standardized, jaw-stratified descriptive analyses were performed. These analyses were not used for confirmatory inference because of small cell counts. The direction of the observed day 0 to day 56 change favored plasma-treated groups in both the maxilla and mandible, but residual confounding from local bone quality, implant dimensions, and site conditions cannot be excluded (Table 5).

**Table 5:** Jaw-stratified descriptive ISQ analysis

Group	Jaw	*n*	Day 0 ISQ	Day 56 ISQ	Day 56 - Day 0 change
Control	Maxilla	4	70.2 ± 1.7	71.4 ± 1.9	1.2 ± 3.5
Control	Mandible	6	70.9 ± 1.4	72.9 ± 1.6	2.0 ± 2.7
Vacuum Plasma	Maxilla	5	71.3 ± 1.7	82.6 ± 1.4	11.3 ± 3.0
Vacuum Plasma	Mandible	5	70.5 ± 1.6	82.0 ± 1.5	11.5 ± 2.5
Argon Jet Plasma	Maxilla	5	70.7 ± 1.3	81.1 ± 1.8	10.4 ± 2.6
Argon Jet Plasma	Mandible	5	70.7 ± 1.4	81.3 ± 1.8	10.6 ± 2.9
Cold Atmospheric Plasma	Maxilla	4	70.7 ± 1.9	81.1 ± 1.7	10.4 ± 3.5
Cold Atmospheric Plasma	Mandible	6	70.5 ± 1.2	80.3 ± 1.5	9.8 ± 1.9

Values are mean +/- standard deviation. Cell sizes are small; therefore, these data are descriptive and not confirmatory subgroup analyses

### Sensitivity analyses for site-level confounding

Sensitivity analyses were performed to address the concern that variables reported in Table 1 may have influenced the primary endpoint. In the conservative one-control-implant deletion series, the main LMM was refitted after removing each possible control implant in turn. The direction of the results remained unchanged in all 10 deletion models. The group-by-time interaction remained significant in every model (chi-square range, 174.69-188.64; all *p* < 0.001). The smallest retained day-56 plasma-control differences across the deletion series were + 9.65 ISQ units for vacuum plasma, + 8.55 for argon jet plasma, and + 7.95 for cold atmospheric plasma. Thus, the observed direction was not dependent on any single control implant, including the control implant with undersized osteotomy (Table 6).

**Table 6:** Sensitivity mixed-effects analyses addressing site-level confounding

Analysis	Model / sample	Group-by-time result	Day-56 plasma-control result
Main LMM	160 observations; group, time, and group x time; patient random intercept; implant nested within patient variance component	chi-square(9) = 188.73, *p* < 0.001	+ 10.0 Vacuum; +8.9 Argon Jet; +8.3 CAP
One-control-implant deletion series	10 ML LMMs, each with one control implant removed (156 observations/model); conservative check that necessarily includes the single undersized control implant	chi-square range = 174.69-188.64; all *p* < 0.001	Minimum retained differences: +9.65 Vacuum; +8.55 Argon Jet; +7.95 CAP
Jaw-adjusted exploratory LMM	Main ML LMM plus jaw location as a fixed effect; sensitivity only, not primary adjusted analysis	chi-square(9) = 188.44, *p* < 0.001; jaw beta = 0.009, *p* = 0.984	+ 10.0 Vacuum; +8.9 Argon Jet; +8.3 CAP

The one-control deletion series is reported as a conservative robustness check because undersized osteotomy occurred only once and only in the control group

*LMM* linear mixed-effects model, *ML* maximum likelihood, *CAP* cold atmospheric plasma

In the exploratory jaw-adjusted LMM, adding jaw location as a fixed effect did not materially alter the findings. The jaw fixed effect was negligible (maxilla vs. mandible beta = 0.009 ISQ units, *p* = 0.984), the group-by-time interaction remained significant (chi-square(9) = 188.44, *p* < 0.001), and the day-56 plasma-control contrasts remained essentially unchanged (+ 10.0, + 8.9, and + 8.3 ISQ units for vacuum plasma, argon jet plasma, and cold atmospheric plasma, respectively). These analyses reduce concern that the main result was driven solely by the single undersized control implant or by jaw distribution; however, they remain exploratory and do not eliminate residual confounding from local bone quality, implant dimensions, or site conditions.

LMM, linear mixed-effects model; ML, maximum likelihood; CAP, cold atmospheric plasma. The one-control deletion series is reported as a conservative robustness check because undersized osteotomy occurred only once and only in the control group.

## Discussion

The present randomized within-patient clinical trial found that immediate pre-insertion cold plasma surface activation was associated with a more favorable observed early ISQ trajectory than untreated control implants during the first 56 days after placement. The main signal of the study was not a difference in insertion-time mechanical stability, because baseline ISQ values were closely comparable among the four groups. Rather, the difference emerged during the early healing period: untreated control implants showed a transient decrease in mean ISQ, whereas all plasma-treated groups showed increasing mean ISQ values over time. This pattern should be interpreted as a difference in early clinical stability trajectory, not as direct proof of histologic osseointegration. ISQ is a clinically useful, non-invasive, and repeatable surrogate measure, but it does not directly quantify bone-to-implant contact, peri-implant bone level, histologic bone formation, or long-term functional success [1].

From a clinical perspective, the attenuation of early stability loss is noteworthy. ISQ values around 70 or higher are generally interpreted as high stability [1]. In the present study, the control group decreased to the moderate-stability range at day 28, whereas all plasma-treated groups remained above 74 ISQ units. At day 56, all groups returned to or remained within the high-stability range, but plasma-treated implants still showed approximately 8–10 ISQ units higher model-based values than untreated controls. This magnitude appears clinically relevant as an early stability signal, particularly because the 2- to 4-week healing window corresponds to the period in which the transition from mechanical primary stability to biologically mediated secondary stability is most critical [4, 5]. Nevertheless, because no implant was functionally loaded and no long-term prosthetic outcome was assessed, these findings should not be used to justify changes in early-loading protocols without dedicated loading studies [3].

The observed pattern is consistent with the limited but growing clinical literature on plasma-treated implant surfaces. Kim et al. reported that plasma-treated implants showed continuously increasing ISQ values over an 8-week period without an obvious early decrease [30]. Similarly, Stacchi et al. reported improved early stability outcomes after vacuum plasma activation in a single-blind split-mouth randomized clinical trial [31]. The present trial extends this clinical context by evaluating three chairside plasma activation approaches within the same patient cohort. Although the study was not designed to establish superiority among plasma systems, the consistency of the control-versus-plasma separation across all treated groups supports the concept that immediate pre-insertion plasma activation may favor early bone-implant interaction during the vulnerable early healing phase. In the broader field of oral implantology, argon plasma treatment has also been investigated for improving the osteoconductive behavior of bone grafting materials [29], while systematic reviews have emphasized the biological potential of plasma-mediated titanium surface activation [13, 14, 33–36].

Several biological mechanisms may explain the observed clinical trajectory, although they remain indirect in the present study because no surface analytical or cellular experiments were performed. Titanium implant surfaces undergo biological aging through atmospheric hydrocarbon accumulation, which reduces surface energy and hydrophilicity and may impair early protein adsorption and osteogenic cell attachment [9–11]. Plasma activation has been shown experimentally to reduce hydrocarbon contamination, increase oxygen-containing surface groups, improve hydrophilicity, and enhance surface energy [18–20, 33–41]. These physicochemical changes may facilitate adsorption of blood- and tissue-derived proteins, osteoblast adhesion, spreading, and early osteogenic differentiation [19, 20, 33, 36, 39]. Preclinical studies have also reported increased bone-to-implant contact and bone volume around plasma-treated titanium implants [18, 25–28, 35], and human histologic evidence suggests that plasma-treated implant surfaces may achieve increased bone-to-implant contact during early healing [17]. Therefore, the present ISQ findings are biologically plausible, but the absence of SEM, XPS, contact-angle measurement, protein adsorption assays, or histologic sampling prevents direct mechanistic confirmation.

Potential antimicrobial and soft-tissue-related effects of plasma activation also deserve consideration, but they should not be overextended in interpreting the present data. Experimental studies have reported biofilm reduction on titanium and other oral biomaterials after cold plasma application [21–24], and cold atmospheric plasma has also been investigated in relation to peri-implant decontamination and regenerative membrane surfaces [24, 28, 37]. In addition, argon plasma pretreatment of healing abutments has been associated with changes in peri-implant microbiome and soft-tissue integration in a proof-of-concept randomized study [42]. However, the present trial did not evaluate microbiological outcomes, peri-implant soft-tissue parameters, inflammatory markers, or patient-centered outcomes. Thus, any antimicrobial or soft-tissue interpretation remains speculative and secondary to the primary ISQ-based finding.

The comparison among the three plasma systems should be interpreted cautiously. Vacuum plasma and argon jet plasma showed numerically higher mean ISQ values than cold atmospheric plasma at day 28, whereas adjusted plasma-to-plasma differences were no longer statistically significant at day 56. This suggests that the clinically relevant late early-healing pattern was broadly similar across the three plasma-treated groups. However, the trial was primarily structured to compare plasma-treated implants with untreated controls, not to prove superiority of one plasma system over another. In addition, differences among plasma systems may depend on treatment atmosphere, discharge characteristics, exposure uniformity, gas composition, pressure conditions, and the degree of surface decontamination or hydrophilicity achieved [40, 41]. Because these properties were not measured in the present study, any explanation that one system produced stronger or more uniform surface activation would be speculative. From a practical standpoint, the current findings suggest that all three technologies may be warrant further clinical investigation, while future comparative trials should incorporate surface characterization to determine whether device-specific physicochemical differences translate into clinically meaningful differences.

A central methodological issue is the nature of the within-patient design. Having each patient contribute one implant to each treatment arm reduced host-level biological variability and improved internal comparability at the patient level. However, the design was not a strict mirrored-site or contralateral split-mouth model. Implant positions were determined according to prosthetic rehabilitation needs, and therefore implant sites could differ in jaw location, local bone density, cortical thickness, implant dimensions, extraction history, and site-specific surgical conditions. Similar baseline ISQ values indicate comparable insertion-time mechanical stability, but they do not establish full anatomical or biological equivalence of implant sites. This distinction is important because jaw location and implant dimensions are known to influence primary stability and healing behavior [43, 44]. Accordingly, the observed plasma-control association should be interpreted as promising but not fully protected from site-level confounding.

The undersized osteotomy issue should be framed within this same methodological context. Undersized osteotomy was used in only one control implant and was not used in any plasma-treated implant. Because undersized preparation is intended to increase mechanical anchorage, its occurrence in the control group would be expected, if anything, to favor control stability rather than inflate plasma-group performance. Additional sensitivity analyses were therefore reassuring: the direction of the plasma-control association remained unchanged after deleting each control implant one at a time, including the undersized control implant, and the exploratory jaw-adjusted LMM did not materially alter the day-56 plasma-control contrasts. These analyses reduce the likelihood that the main finding was driven solely by a single control implant or by jaw distribution. Nevertheless, these analyses do not eliminate residual confounding from local bone quality, cortical architecture, implant dimensions, or other site-specific variables. In particular, implant length distribution was described in Table 1 but was not formally adjusted in the primary model because of the small patient-level sample and sparse cells; therefore, residual confounding related to implant dimensions cannot be excluded. Accordingly, the sensitivity analyses should be interpreted as robustness checks rather than definitive covariate-adjusted evidence of causality.

Patient-level factors also require careful interpretation. Heavy smokers were excluded, but light smokers were present in the enrolled cohort. Because each analyzed patient contributed one implant to each treatment arm, smoking exposure was balanced across groups at the implant level. However, the patient-level sample was too small for meaningful smoking-adjusted modelling, and contemporary evidence indicates that smoking is associated with increased risk of early implant failure [45]. Similarly, medically compromised conditions such as hyperlipidemia, metabolic syndrome, type 2 diabetes mellitus, and chronic disease-related changes in blood-surface interactions may influence osseointegration or implant-surface wettability [46–48]. These conditions were not directly evaluated in the present study. Therefore, no inference can be made regarding the independent effect of plasma activation in smokers or medically compromised patients.

Several limitations should be acknowledged. First, the final analyzed sample was small, with 10 patients and 40 implants, and the effective inferential sample was closer to the number of patients than to the raw implant count. Second, the trial was retrospectively registered, which reduces prospective transparency even though the current manuscript clarifies the implemented design. Third, implant sites were clinically indicated rather than anatomically mirrored, and residual heterogeneity related to jaw location, bone quality, implant dimensions, extraction history, and surgical site conditions cannot be excluded. Fourth, the operating surgeon could not be blinded, complete participant blinding was not feasible because no sham plasma procedure was used, and formal intra-observer repeatability statistics for ISQ measurement were not prospectively recorded. Fifth, the study did not include surface characterization, histologic analysis, radiographic marginal bone-level assessment, inflammatory or microbiological endpoints, functional loading outcomes, or long-term follow-up. These limitations restrict causal inference and clinical generalizability.

Despite these limitations, the study provides preliminary comparative clinical data on three chairside cold plasma activation strategies evaluated against untreated controls within the same patient cohort. The consistent separation between untreated and plasma-treated ISQ trajectories during the first 8 weeks supports further investigation of plasma activation as a potential surface bioactivation strategy. Future studies should be prospectively registered, multicenter, adequately powered at the patient level, and ideally designed with mirrored or site-standardized allocation. They should also include preplanned adjustment or stratification for jaw location, implant dimensions, bone density, smoking status, and systemic risk factors; surface analytical validation; radiographic or histologic bone-response outcomes; and post-loading follow-up. Until such evidence is available, the present findings should be regarded as hypothesis-generating evidence of a favorable early stability trajectory rather than definitive evidence of enhanced osseointegration, early-loading readiness, or long-term clinical benefit.

## Conclusions

Cold plasma surface activation was associated with higher mean early ISQ values and a more favorable observed early stability trajectory than untreated control implants during the first 8 weeks after placement. Within the limitations of a small, retrospectively registered, site-heterogeneous, within-patient trial, vacuum plasma, argon jet plasma, and cold atmospheric plasma all showed favorable control-versus-plasma separation, while plasma-to-plasma differences remained exploratory. The findings should not be interpreted as direct evidence of enhanced osseointegration, early-loading readiness, or long-term clinical benefit. Larger prospectively registered, site-standardized randomized trials with surface characterization, radiographic or histologic outcomes, post-loading follow-up, and patient-level power calculations are required before definitive clinical recommendations can be made.

## Supplementary Information

Supplementary Material 1.

## Data Availability

The datasets generated and/or analyzed during the current study are available from the corresponding author upon reasonable request.

## Abbreviations

AIC: Akaike Information Criterion
ANOVA: Analysis of Variance
BIC: Bone-to-Implant Contact
CAP: Cold Atmospheric Plasma
CONSORT: Consolidated Standards of Reporting Trials
ICC: Intraclass Correlation Coefficient
ISQ: Implant Stability Quotient
LMM: Linear Mixed-Effects Model
ML: Maximum Likelihood
RFA: Resonance Frequency Analysis
XPS: X-ray Photoelectron Spectroscopy
